# Maternal group B Streptococcus recto vaginal colonization increases the odds of stillbirth: evidence from Eastern Ethiopia

**DOI:** 10.1186/s12884-018-2044-2

**Published:** 2018-10-19

**Authors:** Tesfaye Assebe Yadeta, Alemayehu Worku, Gudina Egata, Berhanu Seyoum, Dadi Marami, Yemane Berhane

**Affiliations:** 1 School of Nursing and Midwifery, College of Health and Medical Sciences, Haramaya University, Harar, Ethiopia; 2 Department of Epidemiology and Biostatistics, School of Public Health, Addis Ababa University, Addis Ababa, Ethiopia; 3 School of Public Health, College of Health and Medical Sciences, Haramaya University, Harar, Ethiopia; 4 Department of Medical Laboratory Sciences, College of Health and Medical Sciences, Haramaya University, Harar, Ethiopia; 5 Department of Epidemiology, Addis Continental Institute of Public Health, Addis Ababa, Ethiopia

**Keywords:** Stillbirth, GBS, Maternal recto vaginal colonization, Harar, Ethiopia

## Abstract

**Background:**

Group B *Streptococcus* (GBS) causes a significant number of stillbirths. Despite this, there is little documented information on the association between stillbirth and pregnant women’s GBS recto vaginal colonization in Sub Saharan Africa. As such, this study was aimed at identifying the association between stillbirth and pregnant women’s GBS recto vaginal colonization in Eastern Ethiopia.

**Methods:**

A health facility-based cross-sectional study was conducted among 1688 pregnant women who came for delivery service in Harar town, Eastern Ethiopia between June to October in 2016. Data were collected using a pre-tested structured questionnaire and checklist (which utilize clinical record). Group B *streptococcus* positivity of the pregnant women was confirmed by culture of recto vaginal swab using selective media. The association between GBS colonization and stillbirth was examined using multivariable logistic regression analysis. A statistical significance was declared at *p*-value ≤0.05.

**Results:**

Of the 1688 pregnant women who participated in the study, 144 had stillbirths, representing a prevalence of 8.53% [(95% CI: (7.19, 9.86)]. Group B *Streptococcus* colonization at birth was detected in 231 women (13.68%; 95% CI 12.04, 15.32). Of these 144 stillbirths 59 (40.97%) were from colonized mothers and 72(59.03%) were from non-colonized mothers. Of these 59 stillbirth from colonized mothers, 32(54.23%) were intrapartum stillbirth, 27(45.77%) were antepartum stillbirth occur before exposed to intrapartum antibiotic prophylaxis (IAP). After controlling for potential confounders, the odds of having a stillbirth were 8.93 times higher among recto vaginal GBS colonized pregnant women [AOR = 8.93; 95% CI; (5.47, 14.56)].

**Conclusions:**

This study demonstrated a significant association between maternal recto vaginal GBS colonization and stillbirth. Efforts to reduce stillbirth need to consider prevention of GBS colonization among pregnant women. Maternal vaccination may provide a feasible strategy to reduce stillbirth due to GBS.

## Background

The prevention of stillbirth remains a major global challenge. Of the estimated 2·6 million stillbirths that occur globally every year, 98% occur in low-income and middle-income countries [1]. Efforts to reduce stillbirths in low-income countries have not shown much progress. Sub-Saharan Africa harbors the highest stillbirth rates with Ethiopia ranking fifth among the ten countries with the highest stillbirth rates in the world [1, 2].

Stillbirth is a major cause of psychosocial distress, grief and guilt to families [3]. In Sub Saharan Africa, mothers who have stillbirth are often subjected to severe psychosocial pressure; especially women who experience repeated stillbirth, can be out casted, dishonored and subjected to divorce [4, 5].

The causes of stillbirth vary greatly from country to country. In low income countries about one-third of all stillbirths are attributed to maternal infection during pregnancy [6]. Group B *Streptococcus* colonization and vertical transmission can cause fetal infection and stillbirth as a direct result of toxin-induced cytolysis [7, 8]. Group B *Streptococcus* is estimated to account for 15% of all infection related stillbirths globally [9] and Africa harbor 73.68% of the estimated GBS caused stillbirth [10]. Other factors associated with stillbirth include: higher maternal age, residence, lower economic status, lower education level, poor antenatal care (ANC) uptake, antepartum hemorrhage, essential hypertension, pre-eclampsia, obstetric complication during labour, preterm delivery, low birth weight, and fetal mal-presentation [11, 12].

Stillbirths due to GBS are preventable [9, 13]. However, most of Sub-Saharan African countries do not have guidelines for the prevention of GBS infection, which could increase associated health risks to the mother and fetus [14]. The inaction could be related to a lack of evidence from the region. As such, this study assessed the association between GBS colonization and stillbirth in Eastern Ethiopia.

## Methods

### Study setting

This study was performed in three health facilities (Hiwot Fana Specialized University Hospital, Jugal Hospital and Arategna Health Center) in Harar town, Eastern Ethiopia. Harar is located 510 km east of the capital city of Ethiopia, Addis Ababa. Ethiopia is a low-income country with a total fertility rate of 4.6 per woman [15], stillbirth rates of 25.5 per 1000 birth [11], a neonatal mortality rate of 29 per 1000 live births [15] and 34.3% of neonatal deaths are due to infection [16].

### Study design and sampling

A cross-sectional study was done between June and October 2016. The study participants were pregnant women came for delivery services at labour and delivery rooms of the selected health facilities. This study is part of a larger study conducted to assess GBS colonization among a total of 1688 singltone pregnant women. Women who recieved antibiotics in the last 2 weeks during their pregnancy prior to specimen collection and women who came due to vaginal bleeding prior to labour began, were excluded from this study [17, 18]. Study participants were taken proportional to the client load of the selected health facilities.

### Data collection

Data were collected using a pretested and structured questionnaire. In addition, a checklist was used to extract data from the medical record, and a laboratory test was performed to detect GBS colonization from a recto-vaginal swab. The data collection tools were developed by reviewing previous related data collection instruments [15, 19]. The original questionnaire was prepared in English language and later translated into the local languages (Amharic and Afan Oromo) for data collection. Forward and backward translations were performed by language experts.

A six-day training was given to attending midwives and the supervisors who worked as data collectors. The training for data collectors and supervisors addressed issues related to data collection procedures and tools, interviewing techniques, medical record review and specimen collection procedures. Two medical microbiologists were read, confirm and interpret laboratory test results. Three medical laboratory technologists involved in media preparation and sterilization processes. A research manual was prepared to guide training, data collection and management.

### Measurement

Stillbirth was the dependent variable in this study and this information was obtained from medical records. It was labelled as “yes” and “no” with a code of 1 and 0, respectively. ANC follow-up, hypertensive disease, women’s GBS test result, prolonged labour, birth weight and gestational age were independent variables.

Age of the mother was documented based on maternal reply and later grouped as 15–19, 20–24, 25–29 and 30–34 and 35+ with code 1, 2, 3, 4 and 5, respectively for analysis. Maternal education was grouped as “literate” for those who could read and write and all others as “illiterate” and was coded 0 and 1, respectively. Number of births (parity) were grouped as one primiparity (one delivery), multiparous (2–4 delivery) and grand multiparous (≥ 5 deliveries) and code with 1, 2 and 3, respectively [20]. To determine the economic status of the families, wealth index was used. The wealth distribution was generated by applying principal components analysis to 33 household variables. The wealth status was determined by using three groups, poor as 1, middle as 2, and better as 3 [21].

Hypertesive disease and prolonged labour were obtained from the medical record and labeled as “yes” and “no” and coded with 1 and 0, respectively. Birth weight was categorized as < 2500 g and ≥ 2500 g and labeled as “yes” and “no” and code with 1 and 0, respectively. Similarly, gestational age was categorized as < 37 weeks and ≥ 37 weeks and labeled as “yes” and “no” and code with 1 and 0, respectively.

The specimens were collected by the attending midwives from the lower third of the vagina and rectum of women using a sterile cotton applicator. The specimens collection were performed at admission to the labour and delivery room of the selected health facilities. The laboratory procedure was done following the Center for Disease Control and Prevention (CDC) recommendation and Standard Operating Procedures described elsewhere [22, 23]. Details of specimen transportation and processing have been published previously [24]. A positive GBS reading was labeled as “yes” and a negative reading was labeled as “no”.

### Statistical analysis

Initially crude odds ratio (COR) along with 95% confidence interval was estimated to assess the association between each independent variable and the outcome variable. Variables with *p*-value ≤0.20 in the bivariable analyses were considered in the multivariable logistic regression model. Important variables deemed to be considered were also included, though they did not reach a *p*-value less than 0.20. Multicollinearity was tested using the Variance Inflation Factor (VIF) test, the tolerance test and values of the standard error. No multicollinearity problem was found. Correlation matrix and covariate matrix were tested for the final model. The final model selections were performed by: log likelihood ratio test, Akaike’s information criterion, and Bayesian information criterion. The Hosmer-Lemeshow goodness-of-fit tests were used to test for model fitness [25]. The logistic regression model was used to assess the association between women’s GBS recto vaginal colonization and the outcome variable (stillbirth) by controlling for other potential confounding variables. Adjusted Odds Ratio (AOR) along with 95% confidence interval was estimated to assess the strength of the association. Statistical significance was declared at a *p*-value ≤0.05.

### Ethical approval and consent to participate

The study material was reviewed and approved by the Institutional Health Research Ethics Review Committee of the College of Health and Medical Sciences at Haramaya University. permission was obtained from each of the health facilities involved in the study. Women detected to have GBS colonization were provided antibiotic prophylaxis and women who had a stillbirth received counseling. Neonates with health problems were treated and immediate referral was also facilitated.

## Results

The study enrolled a total of 1688 pregnant women. The age of participants ranged from 15 to 46 years with the mean (standard deviation) of 26.59 (± 5.63) years. Almost all (95.73%) of the participants were married, 47.04% had no formal education, and 44.14% were rural residents (Table 1). A large proportion (33.23%) of the pregnant women had never attended ANC at a health facility. The mean number of deliveries was 2.6 (with a range from 1 to 14). About 11 % of pregnant women had experienced hypertension, 23.22% had experienced prolonged labour, and 15.26% had low birth weight. Group B *Streptococcus* colonization at birth was detected in 231 (13.68%; 95% CI 12.04, 15.32) (Table 2).

**Table 1:** Socio-demographic characteristics of the study participants Harar town, Eastern Ethiopia, 2016

Characteristics	Frequency (*n*)	Percent (%)
Residence of mothers		
Urban	943	55.86
Rural	745	44.14
Age of mothers in year		
≤ 19	301	17.83
20–24	510	30.21
25–29	461	27.31
30–34	275	16.29
≥ 35	141	8.35
Ethnicity		
Oromo	1214	71.92
Amhara	267	15.82
Harari	95	5.63
Somali	15	0.89
Other^a^	97	5.75
Religion		
Muslim	1286	76.18
Orthodox christian	308	18.25
Protestant	89	5.27
Catholic	5	0.3
Marital status		
Married	1616	95.73
Others^b^	72	4.27
Educational status		
Literate	894	52.96
Illiterate	794	47.04

Others^a^ Gurage, Tigre, Silte,…; others^b^ single, separated, divorced, widowed

**Table 2:** Pregnancy, labour, and delivery related characteristics of the study participants Harar town, Eastern Ethiopia, 2016

Characteristics	Frequency (*n*)	Percent (%)
Para		
1	722	42.77
2–4	755	44.73
≥ 5	211	12.50
ANC follow up		
No	561	33.23
Yes	1127	66.77
Anemia		
No	1535	90.94
Yes	153	9.06
Hypertensive disease		
No	1502	88.98
Yes	186	11.02
Prolonged labour		
No	1296	76.78
Yes	392	23.22
Birth weight		
≥ 2500 g	1422	84.24
< 2500 g	266	15.76
Gestational age		
≥ 37 weeks	1540	91.23
< 37 weeks	148	8.77
GBS test result		
Negative	231	13.68
Positive	1457	86.32
IAP received		
No	964	57.11
Yes	724	42.89

*IAP* intrapartum antibiotic prophylaxis

The overall, 144 had stillbirths, representing a proportion of 8.53% [(95% CI: (7.19, 9.86)]. Of these 144 stillbirths 59 (40.97%) were from colonized mothers, 72(59.03%) were from non-colonized mothers. Of these 59 stillbirth from colonized mothers, 32(54.23%) were intrapartum stillbirth, 27(45.77%) were antepartum stillbirth occur before exposed to IAP (Table 3).

**Table 3:** Factors associated with stillbirth among pregnant women, Harar, Eastern Ethiopia, 2016

Characteristics	Stillbirth	Live birth	Crude OR			Adjusted OR		
	*n* = 144(%)	*n* = 1544(%)	OR	95% CI		OR	95% CI	
GBS colonization								
No	5.83	94.17	1			1		
Yes	25.54	74.46	5.53	3.83	7.99	8.93***	5.47	14.56
ANC follow up								
No	15.15	84.85	1			1		
Yes	5.24	94.76	0.3	0.21	0.43	0.53**	0.34	0.82
Anemia								
No	7.88	92.12	1			1		
Yes	15.03	84.97	2.06	1.27	3.34	0.79	0.42	1.45
Hypertensive disorder								
No	6.32	93.68	1			1		
Yes	26.34	73.66	5.29	3.59	7.79	4.66***	2.77	7.84
Prolonged labour								
No	6.10	93.90	1			1		
Yes	16.58	83.42	3.06	2.15	4.34	3.65***	2.38	5.60
Para								
1	8.17	91.83	1			1		
2–4	8.21	91.79	1.00	0.69	1.45	1.46	0.88	2.42
≥ 5	10.90	89.10	1.31	0.82	2.28	1.29	0.58	2.92
Age of women								
≤ 19	10.63	89.37	1			1		
20–24	6.86	93.14	0.6	0.37	1.02	1.11	0.60	2.05
25–29	9.33	90.67	0.86	0.53	1.4	1.10	0.58	2.11
30–34	5.82	94.18	0.51	0.27	0.96	0.42	0.19	0.95
≥ 35	12.77	87.23	1.23	0.66	2.27	0.76	0.30	1.92
Resident								
Urban	5.20	94.80	1			1		
Rural	12.75	87.25	2.66	1.86	3.81	1.27	0.69	2.33
Educational status								
literate	6.26	93.74	1			1		
illiterate	11.08	88.92	1.86	1.31	2.64	1.13	0.69	1.83
Wealth status								
Poor	14.03	85.97	1			1		
Middle	7.00	93.00	0.46	.30	0.68	1.06	0.58	1.91
Better	4.45	95.55	0.28	0.17	0.45	0.65	0.29	1.44
Birth weight								
≥ 2500 g	5.98	94.02	1			1		
< 2500 g	22.18	77.82	4.48	3.11	6.44	1.81*	1.09	3.03
Gestational age								
≥ 37 weeks	5.97	94.03	1			1		
< 37 weeks	35.14	64.86	8.52	5.72	12.69	4.24***	2.41	7.45
IAP received								
No	7.37	92.63	1			1		
Yes	8.53	91.47	1.41	1.00	1.98	1.01	0.67	1.51

*IAP* intrapartum antibiotic prophylaxis, *OR* odds ratio, *CI* confidence intervals; * = *p*, 0.05; ** = *p*, 0.01; *** = *p*, 0.001

The multivariable logistic regression analysis revealed the odds of stillbirth was 8.93 times higher among recto vaginal GBS colonized pregnant women compared to those who had no colonization [AOR = 8.93; 95% CI; (5.47, 14.56)]. In addition, the odds for pregnant women who attended ANC follow-up was lower by 47% [AOR =0.53; 95% CI;(0.34, 0.82)]. The odds for pregnant women with hypertensive disorders was AOR = 4.66; 95% CI; (2.77, 7.8), for those with prolonged labor was AOR = 3.65; 95% CI: (2.38, 5.60), for those with low birth weight was AOR = 1.81; 95% CI: (1.09, 3.03), and preterm delivery AOR = 4.24; 95% CI: (2.41, 7.45) (Table 3).

## Discussion

This study showed a strong association between stillbirths and maternal GBS colonization, even after controlling for other potential confounders. Other studies have also reported similar findings [26, 27]. It is evident that GBS cytolysis breaches maternal-fetal barriers ultimately causing intrauterine fetal death [7]. The stillbirths due to recto vaginal colonization of pregnant women are due to ascending infection. The genome sequencing studies have demonstrated the GBS isolated at birth from the skin of newborns were genetically identical to maternal GBS colonizing isolates [26].

Intrapartum antibiotic prophylaxis is highly effective in preventing newborn’s GBS colonization [28]. However, there are limitations in the clinical benefit of IAP in reducing stillbirth. Antibiotics, for prophylaxis must be administered before the potential exposure, but stillbirth may occur before labour started [29]. In this study, 45.77% of stillbirths occurred before labour began.

Furthermore, identifying pregnant women who need IAP has been challenging for lower- and middle-income countries since most pregnant women did not attend ANC and most deliveries occur at home [30]. However, recent achievements in increasing coverage for the ANC and institutional/ facility delivery could improve the possibility of introducing screening, prevention and treatment interventions in routine ANC and delivery services.

Maternal vaccination could also be cost-effective to prevention programs since it can induce population-level herd immunity, address limitations in the clinical benefit of IAP in reducing stillbirth and late onset of neonatal GBS disease [31, 32]. Maternal immunization could protect the infant and fetus from invasive GBS disease. A clinical trials study, in South Africa, evaluated GBS polysaccharide-protein conjugate vaccine among pregnant women. The vaccine was well tolerated, had similar safety profiles with placebo, and induced capsular-specific antibody responses, in a women. Infant born from vaccinated pregnant women, resulted in higher GBS serotype-specific antibody concentrations [31, 33].

The burden of stillbirth on families, especially women, is severe and long lasting, yet stigma and taboo hinder this burden even in high-income countries. Mothers may be unlikely to seek medical care and such stillbirths could go unreported, thereby skewing or minimizing the actual reported cases of stillbirth [34]. Since women coming to health facilities for delivery could be those with better access to health services and perhaps more aware of the benefits of seeking health services for improved newborn health, the occurrence of stillbirth is likely to be an underestimation. One could also argue those women who opted for institutional delivery could be those with health problems the reported stillbirth represents an overestimation.

Antenatal care follow up was one of the predictors’ of stillbirth. Similar findings were reported in other studies conducted in different part of the world [11, 35]. Despite this, ANC follow up was a protective for GBS related stillbirth. Only 32% of pregnant women had four recommended ANC visits in Ethiopia [15]. To achieve the sustainable development goal to end all preventable stillbirth and neonatal mortality, health promotion programs must better target those communities where maternal health care utilization is low. Urgent effective interventions are therefore needed to improve the ANC coverage as part of a stillbirth and neonatal reduction strategy.

In this study preterm is significantly associated with stillbirth, and this was consistent with previous study [35]. Furthermore, GBS ascending infection, resulting in infection of fetal membranes, decidua and fetus, causing premature rupture of the membrane. Prolonged exposure of the fetus to maternal bacterial flora without any protective membrane barrier enhances the transmission. Host inflammatory response stimulation of prostaglandin and protease synthesis are increases uterine contractility and results in preterm delivery [36]. Vascular insufficiency in pre-eclampsia reduces blood flow to the placenta, leading to hypoxia of the fetus which may also be associated with stillbirth [37, 38]. In prolonged labour, there is also an increased chance that a fetus will suffer from asphyxia due to umbilical cord compression, placental abruption, maternal low blood pressure and birth trauma. Problems with the placenta pose risks to the fetus, including lack of oxygen and nutrients, which can impair fetal growth and could also lead to low birth weight and preterm delivery [12, 39].

The strengths of this study were that a relatively high number of participants included and the study obtained swab samples from both the rectum and vagina, which help to improve the yield [40]. All swabs were transported in Amie’s transport media to the Medical Microbiology Laboratory within 4 h of collection [41]. Selective and differential media were used to isolate and GBS [23].

Measurement and diagnostic biases were minimized by assigning two medical microbiology experts to measure and read the results. Thus, the procedures used was believed to help to reasonably estimate the association. As limitations, the data obtained from the medical records may be potential sources of biases. This has been minimized through verifying the data by the study team before discharging the mother and newborn. Moreover, collecting of microbiological evidence of invasive GBS infection from a normally sterile site, such as fetal blood from the umbilical cord or from the heart, lung aspirate, cerebrospinal fluid or fetal tissues as recommended [42] were not done due to resource constraints. Moreover, immunologic and placenta investigations were also not possible in this setting. Finally, unaccounted and residual confounding could have had an effect on the association found [43, 44].

## Conclusions

This study identified a strong association between maternal recto vaginal colonization of GBS and stillbirth. Efforts to reduce stillbirth need to consider prevention of colonization by GBS among pregnant women. Further stillbirth reduction efforts need to consider introducing effective prevention strategies and improving the quality of the ANC and intrapartum care. Maternal vaccination may provide feasible strategy to reduce stillbirth due to GBS.

## Abbreviations

ANC: Antenatal care
CDC: Center of disease control and prevention
GBS: Group B streptococcus
IAP: Intrapartum antibiotic prophylaxis
LMICS: Low-and-middle income countries
SDG: Sustainable development goal
SOPS: Standard Operating Procedures
